# Red blood cell transfusion may be more detrimental than anemia for the clinical outcome of patients with severe traumatic brain injury

**DOI:** 10.1186/s13054-019-2470-1

**Published:** 2019-05-27

**Authors:** Santiago R. Leal-Noval, María D. Rincón-Ferrari, Manuel Múñoz-Gómez

**Affiliations:** 1 Critical Care Division, University Hospital ‘Virgen del Rocío’, Unidad de Cuidados Intensivos, 1ª planta, Avda Manuel Siurot s/n, 41013 Seville, Spain; 2 Department of Surgical Specialties, Biochemistry and Immunology, School of Medicine, University of Málaga, campus de Teatinos s/n, 29010 Málaga, Spain

To the Editor,

Gobatto et al. [1] have published a RCT in the Journal comparing a restrictive red blood cell transfusion (RBCT) strategy (hemoglobin threshold 7 g/dL; *N* = 23) with a liberal strategy (hemoglobin 9 g/dL; *N* = 21) in ICU patients sustaining severe traumatic brain injury (TBI). When compared with the liberal RBCT group, the restrictive RBCT group had lower 14-day hemoglobin concentration (8.4 ± 1.0 g/dL vs. 9.3 ± 1.3 g/dL, respectively; *p* < 0.01) (primary objective) and higher in-hospital mortality (30 vs. 5%, respectively; *p* = 0.05), as well as a trend toward worsen neurological status, as assessed by unfavorable Glasgow Outcome Scale (GOS) both at discharge and 6 months after hospital discharge (48% vs 38%, respectively; *p* = 0.20) (secondary objectives). Though the authors consider their secondary outcome analysis as exploratory, they stated that their data were in line with previous publications pointing toward the superiority of the liberal transfusion strategy. Several study limitations were acknowledged, mainly that, at baseline, the restrictive group would be more severely affected than the liberal group, and this could explain part of the reported secondary outcomes [1].

Notwithstanding this, we would like to point out that (1) the mean of the difference between both groups was only 0.9 g/dL, but the sample size was calculated to detect a mean difference of 1.2 g/dL. It seems hardly probable that a hemoglobin difference < 1 g/dL may influence the clinical outcome; (2) the total number of RBC units transfused over the length of hospital stay (pre- and post-randomization) was finally higher in the restrictive group (164 U; 7.1 U/patient) than that in the liberal group (131 U; 6.2 U/patient); (3) the authors speculate that cerebral oxygenation, which was not measured in this RCT, could have been impaired in the restrictive arm, thus explaining the poorer clinical outcome in this group. Assessment of cerebral oxygenation may lead to more efficient RBCT. In a recent RCT [2], we explored the reliability of a NIRS threshold-based protocol for RBCT in a mixed population of moderately anemic, neurocritical patients. Compared with the sole use of a predefined hemoglobin threshold, a NIRS threshold resulted in decreased number of transfused patients (from 71 to 59%; RR 0.83 [95% CI 0.62–1.11]) and transfused units (from 1.5 ± 1.4 to 1.0 ± 0.1; *p* < 0.05), with no differences in the incidence of adverse effects reported during 1-year follow-up. Finally, (4) in a 1-year follow-up study of 309 patients with TBI, we found an independent association between RBCT and unfavorable long-term neurocognitive outcome, suggesting that RBCT may exert a more detrimental effect than anemia [3]. In fact, recent guidelines continue recommending the adoption of a restrictive RBCT strategy [4, 5]. Therefore, pending on confirmation by a larger trial, Gobatto et al.’s study results should be interpreted cautiously.
