# Supplementary material for: Red blood cell transfusion may be more detrimental than anemia for the clinical outcome of patients with severe traumatic brain injury
Source: Crit Care. 2019 May 27;23:189. doi: 10.1186/s13054-019-2470-1 (PMC6535857; doi:10.1186/s13054-019-2470-1)
Supplement: Related texts

# Reply: Authors’ response

**Authors:** André L. N. Gobatto, Milena A. Link, Luiz M. S. Malbouisson

We have read with great interest the letter from Dr. Leal-Noval et al. regarding our paper. They raise several questions we are pleased to provide answers.

In the TRAHT study [1], the mean hemoglobin concentration during the first 14 days after hospital admission was 9.3 ± 1.3 g/dL in the liberal group and 8.4 ± 1.0 g/dL in the restrictive group (*p* < 0.01), giving a mean difference of 0.9 ± 0.2 g/dL. It represents the mean difference in the 14 days’ time point, meaning the difference on a daily basis was higher, especially in the first days after randomization. For example, the mean hemoglobin concentration difference on the 10th day was 1.8 ± 0.4 g/dL (95% CI 1.0–2.6, *p* < 0.01) between liberal and restrictive groups. Furthermore, other randomized controlled trials evaluating two transfusion strategies based on hemoglobin triggers in different populations have found similar hemoglobin differences [6].

The TRAHT study was a randomized clinical trial, meaning the only difference between groups after randomization was the transfusion strategy. After randomization, the patients in the liberal group receive a total of 66 red blood cells (RBCs) units as compared with 35 RBC units in the restrictive group (*p* = 0.02), with a good protocol adherence. However, the sample size was small, which may have led to unbalanced groups. The restrictive group had more pupil alterations and more midline deviation ≥ 5 mm on brain CT, and more patients received blood transfusions before randomization compared to the liberal group 9 (43%) vs. 15 (65%), respectively. Nevertheless, the study groups were statistically well matched at baseline with respect to demographic and clinical characteristics. Although not statistically significant, it might suggest that the restrictive group would be more severely injured than the liberal group and this could explain part of the reported secondary outcomes.

Several experimental, physiological, and observational data point toward an increase in the brain tissue oxygen partial pressure (PbtO2) in anemic neurocritical patients that received RBC transfusion [7]. If these results translate into better patient-centered outcomes, whether PbtO2 could be used to identify the patients which benefit from RBC transfusion remains to be determined.

Anemia as well as RBC transfusion is associated with worse outcomes in TBI patients [8, 9]. However, these data come from observational trials which are deeply susceptible to confusion bias, even after statistical analysis of the data and are particularly influenced by the patients’ severity. The most severe TBI patients are anemic, and the most severe anemic TBI patients receive blood transfusion. Thus, TBI patients receiving blood transfusion would be always associated with worse outcomes in this setting. Therefore, only an adequately powered randomized clinical trial could answer the question if a strategy aimed at correcting anemia by RBC transfusion could benefit severe TBI patients. TRAHT was a pilot trial, aimed at evaluating the feasibility of a randomized clinical trial comparing liberal and restrictive blood transfusion strategies in patients with moderate and severe TBI. Although its secondary analysis in favor of the liberal transfusion strategy was noteworthy, we agree that the study results should be interpreted cautiously.
